# Identification of Factors Contributing to Pathogenic Variability Among Lassa Virus Strains Using the Guinea Pig Model and Reverse Genetics

**DOI:** 10.1093/infdis/jiaf323

**Published:** 2025-06-21

**Authors:** Satoshi Taniguchi, Takeshi Saito, Ruchi Paroha, Cheng Huang, Slobodan Paessler, Junki Maruyama

**Affiliations:** Department of Pathology, The University of Texas Medical Branch, Galveston, Texas, USA; Department of Virology I, National Institute of Infectious Diseases, Tokyo, Japan; Department of Pathology, The University of Texas Medical Branch, Galveston, Texas, USA; Department of Pathology, The University of Texas Medical Branch, Galveston, Texas, USA; Department of Pathology, The University of Texas Medical Branch, Galveston, Texas, USA; Department of Pathology, The University of Texas Medical Branch, Galveston, Texas, USA; Department of Pathology, The University of Texas Medical Branch, Galveston, Texas, USA; Institute for Human Infections and Immunity, The University of Texas Medical Branch, Galveston, Texas, USA

**Keywords:** Lassa virus, pathogenicity, pathogenic mechanisms, animal model, reverse genetics

## Abstract

Lassa virus (LASV) is the causative agent of Lassa fever (LF), a severe hemorrhagic disease with potential for lethal outcomes. Classified as a risk group 4 pathogen, it represents a significant public health threat in endemic regions. Our laboratory previously developed a novel lethal guinea pig model of Lassa fever using the clinical isolate LASV strain LF2384. However, the specific pathogenic factors underlying LF2384 infection in guinea pigs remained unclear. In this study, we aimed to investigate the immunological differences elicited by LF2384 and LF2350, another LASV strain isolated from a nonlethal case within the same outbreak. We compared the expression kinetics of key immune-related genes in guinea pigs infected with either LF2384 or LF2350. Using reverse genetics, we identified the L protein as a critical viral factor responsible for the observed pathogenic differences between the strains.

## Teaser

Using reverse genetics and animal models with 2 Lassa virus (LASV) strains exhibiting contrasting phenotypes, we identified host and viral factors that contribute to LASV pathogenicity. The findings in this study offer insight into the molecular mechanisms underlying LASV infection.

Lassa virus (LASV), a member of the family Arenaviride, is the causative agent of Lassa fever (LF). LF is endemic in West African countries, including Nigeria, Guinea, Liberia, and Sierra Leone. It poses a major public health threat in these regions, with an estimated 500 000 LASV infections annually [1]. The case-fatality rate among hospitalized patients ranges from 15% to 70% [2–4]. Although an estimated 37.7 million people are at risk for LF, available countermeasures against LASV remain extremely limited [5]. Due to its high pathogenicity and lack of approved vaccines or therapeutics, LASV must be handled in biosafety level 4 (BSL-4) facilities, creating substantial logistical and resource-related challenges for LF research. Nonetheless, understanding the factors that contribute to LASV pathogenicity is essential for the development of effective countermeasures.

Animal models of LF are limited, particularly those based on clinical LASV. The nonhuman primate model—considered the gold standard for LF studies—closely mirrors lethal human disease [6, 7]. However, its use is constrained by high cost and complex safety concerns associated with high-containment laboratory environments. To address these challenges, our laboratory recently developed a novel guinea pig model of LF using a clinical LASV isolate [8]. In this model, outbred Hartley guinea pigs develop a uniformly lethal infection when infected with LF2384, a clinical LASV isolate from a lethal LF case, without requiring host-virus adaptation [8]. In contrast, the LASV strain LF2350, isolated from a nonlethal LF case during the same outbreak, did not cause lethal infection in guinea pigs. These findings suggest that our novel guinea pig models are suitable for investigating the pathogenic mechanisms of LASV infection, allowing for the identification of pathogenic factors through a combination of molecular and in vivo approaches.

In this study, we examine the host and viral factors contributing to the differential pathogenicity of LF2384 and LF2350. The findings provide insights into the molecular mechanisms underlying LASV infection and support the development of effective preventive and therapeutic strategies against LF.

## METHODS

### Cells and Viruses

Vero, Vero E6, A549, Huh-7, and 104C1 cells were maintained in Dulbecco's modified Eagle medium (DMEM) supplemented with 10% fetal bovine serum (FBS) and 100 U/mL of penicillin-streptomycin. All cells were obtained from the American Type Culture Collection, except for Huh-7 cells, which were obtained from the Japanese Collection of Research Bioresources. BHK-21S cells were maintained in DMEM supplemented with 5% FBS, 10% tryptose phosphate broth (Gibco), 100 U/mL penicillin-streptomycin, and 2 mM L-glutamine. All cell lines were cultured at 37°C in 5% CO_2_.

The LASV strains LF2384 (gene accession number, PP826288 and PP826289) and LF2350 (gene accession number, PP826286 and PP826287), both belonging to lineage IV, were isolated from serum samples of human LF cases during a 2012 outbreak in Sierra Leone [8, 9]. Nucleotide and amino acid sequence differences between the strains are presented in Table 1, and Supplementary Tables 1 and 2. The viruses were propagated in Vero cells from patient serum, and the virus-containing cell culture supernatant was stored in a −80°C freezer until use. All research involving LASVs or recombinant LASVs (rLASVs) was approved by the Institutional Biosafety Committee at The University of Texas Medical Branch (UTMB) and conducted in BSL-4 facilities at the Galveston National Laboratory (GNL), UTMB, following institutional guidelines.

**Table 1.** Amino Acid Differences in Lp Between Lassa Virus LF2384 and LF2350

	Domain						Total
	Endonuclease	Linker	PA-C–Like Region	RdRp	PB2-N–Like Region	C-Terminal	
Number of variant amino acid	5	2	40	34	4	36	121
Putative number of amino acid	194	66	450	902	209	399	2220
Identity, %	97.4	97.0	91.1	96.2	98.1	91.0	94.5

### Virus Titration

Virus titers were determined through plaque assay as previously described [8]. Briefly, confluent monolayers of Vero cells in 12-well plates were inoculated with 100 µL of 10-fold serially diluted virus and incubated for 30 minutes at 37°C in a CO_2_ incubator. After removing the inoculum, each well was overlaid with a minimum essential medium containing 2% FBS, 1% penicillin-streptomycin, and 0.6% tragacanth (Sigma). Following a 5–6-day incubation, cells were fixed with 10% formalin and stained with Crystal Violet. Viral titers were expressed as plaque-forming unit (PFU).

### Virus Challenge in Guinea Pigs

Six- to eight-week-old female Hartley guinea pigs were purchased from Charles River Laboratories. All animals were housed in animal biosafety level 2 (ABSL-2) and ABSL-4 facilities at the GNL at UTMB. All animal studies were reviewed and approved by the Institutional Animal Care and Use Committee at UTMB and conducted in accordance with the National Institutes of Health guidelines. Animal identification and body temperature measurement were conducted using subcutaneously implanted BMDS IPTT-300 transponders and a DAS-8027 transponder reader (Avidity Science). Guinea pigs were intraperitoneally inoculated with diluted viruses in 100 µL of phosphate-buffered saline (PBS). PBS was used as the uninfected control. Inoculation titers of rLASVs were standardized to 10^4^ PFU per animal. The ages of the guinea pigs were evenly distributed across all infection experiments. Guinea pigs were monitored for signs of illness by measuring body weight and temperature. Detailed information regarding clinical scoring and end point criteria is provided in the Supplementary Material.

### Complete Blood Count and Blood Clinical Chemistry

Whole-blood samples were collected by cardiac puncture at 11 days postinfection (dpi) from guinea pigs infected with LF2384 or LF2350. Samples were drawn into EDTA tubes (for complete blood count [CBC]) or lithium heparin tubes (for blood clinical chemistry [BCC]) (Becton Dickinson). CBC analysis was conducted using the VETSCAN HM5 (Zoetis), and BCC was assessed using the VETSCAN VS2 analyzer with Comprehensive Diagnostic Profile (Zoetis) following the manufacturer's instructions.

### Immunological PCR Array

Transcriptional analysis of immunological genes was performed using polymerase chain reaction (PCR) assay, as previously described [10]. Briefly, peripheral blood mononuclear cells (PBMCs) were isolated from blood samples collected at 11 dpi from guinea pigs infected with LASV LF2384 or LF2350 using Ficoll-Paque Premium 1.084 (Sigma). Total RNA was extracted from PBMCs using TRIzol reagent (Thermo Fisher Scientific) and the Roche Cellular RNA Large Volume Kit on the MagNA Pure 96 instrument. cDNA was synthesized using the BioRad iScript system. The immunological PCR array was conducted according to established protocols [11 , 12]. β-actin, eukaryotic elongation factor 1-α (*EEF1A1*), glyceraldehyde-3-phosphate dehydrogenase (*GAPDH*), and hypoxanthine-guanine phosphoribosyltransferase 1 (*HPRT1*) were used as internal reference genes. Gene expression levels were normalized using the mean of these 4 reference genes with the ΔΔCt method. Fold change was calculated by dividing the normalized gene expression of the test sample by that of the control sample.

### Viral Genome Sequencing

Viral genome sequences were determined using Sanger sequencing. Total RNA was extracted from infected Vero cells. The open reading frame (ORF) of each viral protein, along with the intergenic regions, was amplified by reverse transcription polymerase chain reaction (RT-PCR). To determine the 5′ and 3′ termini of the viral genome sequence, a 5′ rapid amplification of cDNA ends (RACE) system (Thermo Fisher Scientific) was used, following previously described methods [13]. All amplified DNA fragments were purified, and their nucleotide sequences were analyzed using Sanger sequencing.

### Plasmids

The S- and L-segments of the LASV genome were cloned into the pRF vector, which is driven by a murine RNA polymerase I (mPol-I) promoter and terminator to synthesize viral RNA. An additional G residue—previously reported to enhance the efficiency of virus rescue through reverse genetics—was inserted between the mPol-I promoter and the viral genome sequences [14]. Chimeric L-segment constructs in pRF vectors were generated using the In-Fusion HD Cloning Kit (TaKaRa Bio). Compared to the parent wild-type virus, the LF2384 L protein (Lp) sequence contains 2 synonymous nucleotide substitutions, while the LF2350 glycoprotein (GPC) sequence contains one. Plasmids expressing LASV nucleoprotein (NP; pC-LASV-NP) and Lp (pC-LASV-L) were generated by cloning PCR amplicons of the NP or Lp genes into the pCAGGS expression vector [15]. For the minigenome plasmid (pRF-SMG), the NP gene was replaced with the firefly luciferase (*Fluc*) gene, and the GPC gene was removed based on the pRF vector containing the LASV S segment. A plasmid expressing *Renilla* luciferase (*Rluc*, pRL-SV40) was obtained from Promega.

### Rescue of Recombinant LASVs

The rescue of rLASVs was performed as previously described [13]. Briefly, BHK-21S cells in 12-well plates were transfected using 6.0 µL of FuGENE HD transfection reagent (Promega) with 0.4 µg of pRF-LASV-Sseg, 0.6 µg of pRF-LASV-Lseg, 0.4 µg of pC-LASV-NP, and 0.6 µg of pC-LASV-L. Cells were incubated for 4–5 days. The culture supernatant was then harvested and used to inoculate Vero cells. Supernatants were collected at 5–6 dpi and stored at −80°C. Recombinant strains r2384 and r2350 were passaged twice in Vero cells following their rescue in BHK-21S cells, whereas the other rLASVs were passaged only once. The genome sequences of all recombinant viruses were confirmed as previously described.

### Comparison of Virus Growth

Virus growth kinetics were evaluated as previously described [13 ]. Briefly, Vero E6, Huh-7, A549, or 104C1 cells were infected with each virus at a multiplicity of infection of .001. After a 30-minutes adsorption at 37°C, the cells were washed 3 times, and 1 mL of DMEM supplemented with 2% FBS was added to each well. Culture supernatants were collected daily from 0 to 5 dpi. The supernatants were centrifuged at 8000*g* for 5 minutes to remove cellular debris and stored at −80°C until virus titration by plaque assay.

### Minigenome Reporter Assay

The minigenome reporter assay was performed using a modified version of a previously described protocol [16]. Briefly, BHK-21S cells in 24-well plates were transfected with 0.1 µg of the minigenome plasmid (pRF-SMG), 0.1 µg of pC-LASV-NP, 0.4 µg of pC-LASV-L, and 0.005 µg of pRL-SV40 using FuGENE HD transfection reagent (Promega). At 48 or 72 hours posttransfection, Fluc and Rluc activities were measured using the Dual-Luciferase Assay System and a GloMax 96 luminometer (Promega). Fold induction of *Fluc* expression, normalized to *Rluc* expression, was calculated relative to cells transfected with the empty pCAGGS vector alone.

### Statistical Analysis

Statistical analyses were performed using GraphPad Prism software (version 9.1.2). Fold changes from transcription profiling were analyzed using the ΔΔCt method and Student *t* test. Statistical differences in body weight and temperature were evaluated using a 2-way repeated-measures analysis of variance (ANOVA) followed by Dunnett post hoc test. Viral growth curves were analyzed using a 2-way ANOVA followed by Šidák multiple comparisons test of the log-transformed viral titers. Data from the minigenome reporter assay were analyzed using Student *t* test.

## RESULTS

### Pathogenicity of LF2350 in Guinea Pigs

**Figure 1.**
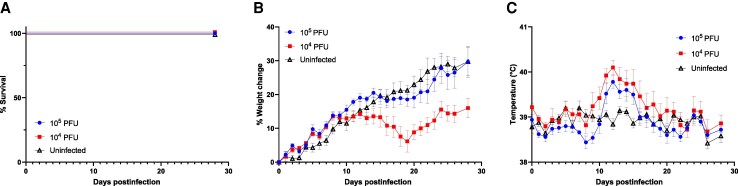
Pathogenicity of LASV LF2350 in Hartley guinea pigs. *A*, Survival rates, *B*, body weight changes, and *C*, body temperatures of Hartley guinea pigs intraperitoneally infected with 10^4^ or 10^5^ PFU of LASV LF2350 (n = 5 per group) or uninfected controls (n = 5) are shown. Error bars represent standard errors of the mean. Abbreviations: LASV, Lassa virus; PFU, plaque-forming unit.

LASV strains LF2384 and LF2350 were isolated from serum samples collected during the 2012 LF outbreak in Sierra Leone. In our previous study, LF2384 caused lethal infection in Hartley guinea pigs [8]. To evaluate the pathogenicity of LF2350, guinea pigs were infected with either 10^5^ or 10^4^ PFU of the virus. Animals infected with 10^4^ PFU exhibited greater weight loss than those infected with 10^5^ PFU. However, the weight loss remained relatively mild compared to that observed in guinea pigs infected with LF2384, which showed 10%–20% reduction from baseline body weight [8]. Transient fever was observed between 10 and 16 dpi. Despite these symptoms, all animals survived without exhibiting overt clinical signs of disease, such as scruffy coat, hunching, or reduced activity, except for transient weight loss and fever (Figure 1). This outcome contrasts sharply with the lethal effects of strain LF2384 [8].

### Hematology and Virus Dissemination of LF2384 and LF2350

**Figure 2.**
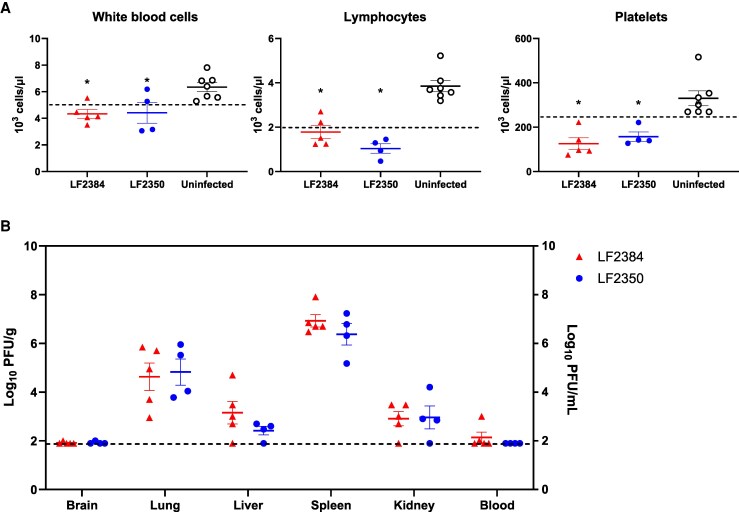
Complete blood counts and viral dissemination in guinea pigs infected with LASVs at 11 dpi. *A*, White blood cell, lymphocyte, and platelet counts from blood samples collected at 11 dpi from guinea pigs intraperitoneally infected with 10^4^ PFU of LASV LF2384 (n = 5), LF2350 (n = 4; 1 animal was excluded due to accidental death immediately after inoculation and before blood sampling), or uninfected control (n = 7). Each symbol represents an individual animal; group means ± SEM are shown. Dashed lines represent the lower limits of the normal range. Statistical analyses were performed using 1-way ANOVA followed by Šidák multiple comparisons test (*P* < .05 vs uninfected controls). *B*, Viral titers in organ and blood samples collected at 11 dpi from guinea pigs infected with 10^4^ PFU of LASV LF2384 (n = 5) or LF2350 (n = 4). Each symbol represents an individual animal; group means ± SEM are shown. The dashed line represents the detection limit (<2.0 log_10_ PFU/g for organs, < 2.0 log_10_ PFU/mL for blood). Abbreviations: LASV, Lassa virus; PFU, plaque-forming unit.

Blood and organ samples were collected at 11 dpi from guinea pigs infected with 10^4^ PFU of either LF2384 or LF2350 to assess CBC, BCC, and virus dissemination (Figure 2, and Supplementary Tables 3 and 4). We chose 11 dpi because guinea pigs infected with LASV exhibited fever and weight loss at this time, which also appeared to coincide with peak virus titers before disease progression (Supplementary Figure 1). Compared to the uninfected controls, guinea pigs infected with either LF2384 or LF2350 showed leukopenia, lymphopenia, and thrombocytopenia. However, no significant differences were observed between the LF2384- and LF2350-infected groups. Similarly, BCC results and virus titers in organs or blood showed no marked differences between the 2 strains.

### Transcription Profiling of Immunological Genes

**Figure 3.**
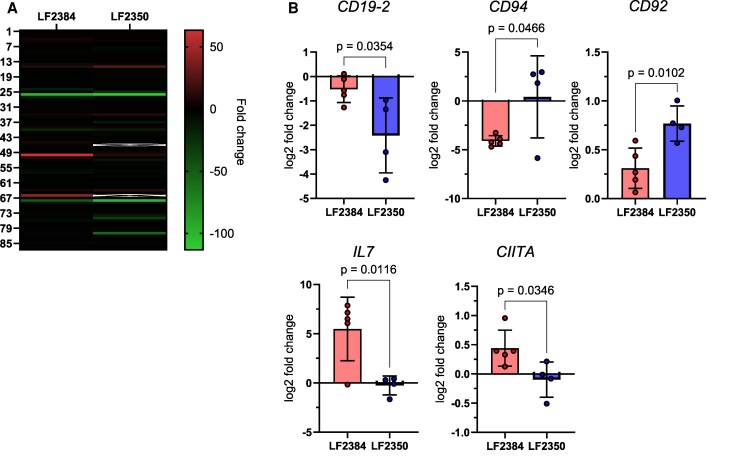
Transcription profiling of immune-related genes in guinea pigs infected with LASVs. *A*, Heatmap showing transcriptional profiles of 87 immune-related genes collected at 11 dpi from guinea pigs infected with LASV LF2384 (n = 5) or LF2350 (n = 4) compared to uninfected controls. Data represent the average fold change in gene expression relative to the uninfected group. *B*, Transcriptional changes in selected genes that showed statistically significant differences between guinea pigs infected with LASV LF2384 (n = 5) and LF2350 (n = 4) at 11 dpi. Fold changes were calculated using the ΔΔCt method, with β-actin, eukaryotic elongation factor 1-α (*EEF1A1*), glyceraldehyde-3-phosphate dehydrogenase (*GAPDH*), and hypoxanthine-guanine phosphoribosyltransferase 1 (*HPRT1*) as internal references. The error bars indicate standard deviations. Statistical significance was determined using unpaired *t* tests; *P* values are indicated. Abbreviations: dpi, days postinfection; LASV, Lassa virus.

PBMCs were collected from guinea pigs infected with 10^4^ PFU of LF2384 or LF2350 at 11 dpi for transcription profiling of 87 immune-related genes (Figure 3). Statistically significant differences (*P* < .05) were observed in 5 transcripts (Figure 3*B*). LF2384 infection resulted in less downregulation of *CD19-2* (a B-lymphocyte antigen) compared to LF2350. LF2384 downregulated *CD94* (a natural killer [NK] cell marker), whereas LF2350 slightly upregulated its expression. *CD92* (choline transporter-like protein 1) was more prominently upregulated following LF2350 infection compared to LF2384. Interleukin-7 (*IL7*) was upregulated in LF2384-infected samples but not in those infected with LF2350. Additionally, the expression of the major histocompatibility complex (MHC) class II transactivator (*CIITA*) was upregulated in LF2384-infected samples and downregulated in LF2350-infected samples.

### Viral Factor Responsible for Pathogenic Differences Between LF2384 and LF2350

**Figure 4.**
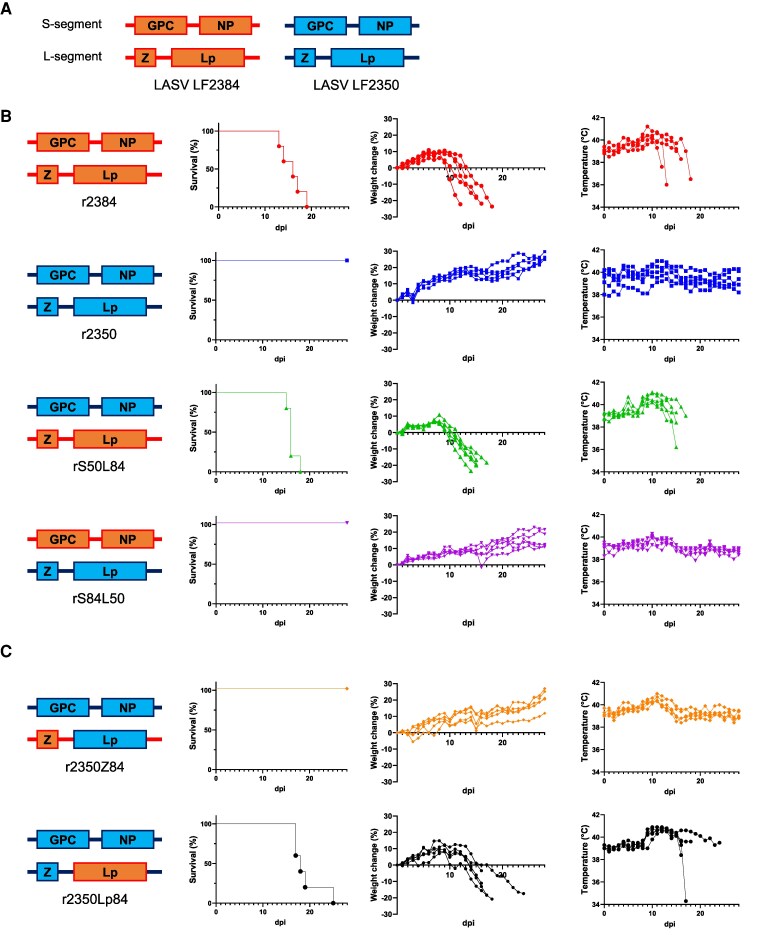
Pathogenicity of rLASVs in guinea pigs. *A*, Schematic representation of rLASVs used in this study. Boxes indicate each ORF of the viral proteins. Lines indicate non-conding regions. *B*, Survival rates, body weight changes, and body temperatures of Hartley guinea pigs intraperitoneally infected with 10^4^ PFU of r2384, r2350, rS50L84, or rS84L50 (n = 5 per group). *C*, Survival rates, body weight changes, and body temperatures of Hartley guinea pigs infected with 10^4^ PFU of r2350Z84 or r2350Lp84 (n = 5 per group). The r2350Lp84 strain contains only the Lp ORF from LF2384, with all other gene segments derived from LF2350. In contrast, r2350Z84 contains the LF2350 S segment and the LF2384 L segment with the Lp ORF from LF2350. Abbreviations: dpi, days postinfection; ORF, open reading frame; PFU, plaque-forming unit; rLASVs, recombinant Lassa virus.

To identify the viral factor underlying the observed differences in LASV pathogenicity, we generated rLASVs and evaluated their pathogenicity in guinea pigs (Figure 4*A*). The rLASVs LF2384 (r2384) and LF2350 (r2350) exhibited pathogenicity comparable to their respective wild-type strains (Figure 4*B*). We also assessed the pathogenicity of segment-swapped rLASVs, rS50L84 and rS84L50. The rS50L84 virus, containing the S segment from LF2350 and the L segment from LF2384, showed pathogenicity similar to that of wild-type LF2384 or r2384. This suggests that viral proteins encoded by the L segment—namely, the Z and/or L protein—are responsible for strain-specific pathogenicity (Figure 4*B*). Subsequently, we generated 2 additional rLASVs: r2350Lp84 and r2350Z84. The r2350Lp84 virus contained the LF2350 S segment and an L segment in which the Lp ORF was derived from LF2384. In contrast, r2350Z84 contained the LF2350 S segment and the LF2384 L segment with the Lp ORF from LF2350. The r2350Z84 was nonpathogenic, similar to r2350, whereas r2350Lp84 caused 100% lethality. These results demonstrate that the LASV Lp is a key determinant of pathogenicity (Figure 4*C*).

### Virus Replication Kinetics and RNA-Dependent RNA Polymerase Function of LASV LF2384 and LF2350

**Figure 5.**
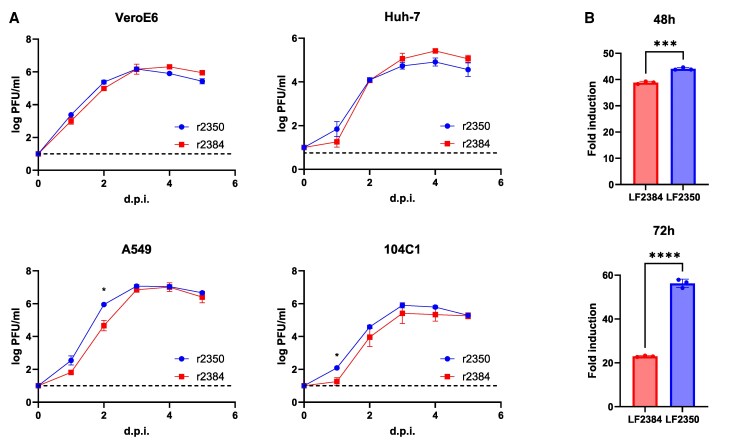
Viral growth kinetics and RdRp activity of LASV LF2384 and LF2350. *A*, Viral replication kinetics of r2384 and r2350 were measured in Vero E6, Huh-7, A549, and 104C1 cells. Data represent the mean and geometric standard deviation from biological triplicates. Statistical analyses were performed using 2-way ANOVA followed by Šidák multiple comparisons test (*P < .05). The dashed lines indicate the detection limits (<10 PFU/mL). *B*, A minigenome reporter assay was used to compare RdRp activity between LASV LF2384 and LF2350. *Fluc* expression was normalized to *Rluc* expression and expressed as fold change relative to cells transfected with pRF-SMG, pC-LASV-NP, and pC-LASV-L of LASV LF2384. Data represent the mean ± standard error from 3 independent experiments. Statistical significance was assessed using Student *t* test (****P < .0001, ***P < .001). Abbreviations: *Fluc*, firefly luciferase; PFU, plaque-forming unit; RdRp, RNA-dependent RNA polymerase; *Rluc*, *Renilla* luciferase.

Because Lp functions as the RNA-dependent RNA polymerase (RdRp), we investigated virus replication kinetics in various cell lines and assessed the RdRp activity. The growth kinetics of r2384 and r2350 were measured in nonhuman primate (Vero E6), human (Huh-7 and A549), and guinea pig (104C1) cell lines, showing no significant differences (Figure 5*A*). These findings are consistent with the in vivo viral dissemination data (Figure 2*B*). However, a minigenome reporter assay revealed that LF2350 exhibited higher RdRp activity than LASV LF2384 (Figure 5*B*), indicating that increased virus growth or RdRp activity is not associated with LASV pathogenicity.

## DISCUSSION

Basic scientific research is essential for developing effective countermeasures against infectious diseases. However, most LASV studies have primarily focused on countermeasure evaluation or in vitro molecular biology, largely due to the requirement that live LASV be handled in BSL-4 facilities. In this study, we investigated host and viral factors involved in LASV pathogenesis using recently developed guinea pig models of LF [8]. We analyzed the differences in host immune responses to LF2384 and LF2350 infections and identified a viral factor contributing to the pathogenicity of LF2384. A comprehensive understanding of LASV pathogenicity from both host and viral perspectives can inform the development of effective countermeasures and help elucidate factors that exacerbate LF in clinical cases.

Despite markedly different disease outcomes, CBC, BCC, and virus dissemination in guinea pigs infected with 10^4^ PFU of LASV LF2384 and LF2350 showed no significant differences at 11 dpi. However, transcriptional profiling of immune-related genes in PBMCs revealed notable differences between the groups. Specifically, *CD94* was downregulated in LF2384-infected guinea pigs but was slightly upregulated in those infected with LF2350, resulting in a 22.8-fold difference. CD94 is expressed in NK cells and a subset of CD8^+^ T cells [17] and plays a critical role in NK-cell–mediated resistance to viral infections [18 ]. Our results suggest that reduced activation of NK cells may contribute to the lethality observed with LF2384 infections. This hypothesis aligns with previous studies indicating that cellular immunity, particularly T-cell–mediated viral clearance, is critical for protection against LASV infection [19–21]. In contrast, early mobilization of NK cells during LF2350 infection may contribute to its low pathogenicity. Furthermore, LF2350-infected animals showed more pronounced downregulation of *CD19-2* (a B cell antigen) compared to those infected with LF2384, indicating a greater suppression of B-cell responses. Upregulation of *IL7* was observed only in guinea pigs infected with LF2384. Because IL-7 is known to support B-cell restoration [22], these findings suggest that B cells decreased in animals infected with both LASV strains but were restored through IL-7 production only in LF2384-infected guinea pigs. While B-cell restoration may contribute to recovery, cellular immunity likely plays a more significant role in determining pathogenicity. Further investigation is required to confirm this hypothesis. The MHC CIITA, which regulates MHC class II expression in antigen-presenting cells [23], has been reported to have either protective or supportive roles in viral infections [24]. Conversely, CIITA has also been reported to induce cellular resistance to viruses that rely on cathepsin-mediated entry, such as the Ebola virus and severe acute respiratory syndrome coronavirus 2 [25]. However, the same study reported that CIITA overexpression did not inhibit infection by vesicular stomatitis virus pseudotyped with LASV glycoprotein. This finding is consistent with our results, which showed that CIITA activation occurred in LF2384-infected guinea pigs despite the infection remaining lethal. Further research is needed to clarify whether CIITA activation contributes to LASV pathogenesis. These findings highlight the complexity of the host immune response to LASV infection and its implications for disease progression. In this study, transcriptional profiling was conducted using PBMC samples collected at 11 dpi, which represents a limitation. This time point was chosen because it coincides with the peak of fever in LASV-infected guinea pigs. Only a few transcriptomic studies on LASV-infected animals have been reported previously [26–28], primarily due to the lack of suitable small animal models for this purpose. Among them, Baillet et al and Hortion et al characterized host immune responses to different LASV strains, such as Josiah and AV [27, 28]. Based on these studies, we chose 11 dpi for sampling, as this time point was associated with peak viral titers, immune gene expression changes, and alterations in CBC/BCC. For example, in LASV Josiah-infected cynomolgus monkeys, samples were collected at peak fever, supporting the relevance of this time point. However, this represents a limitation of the present study. Conducting transcriptomic analyses at multiple time points—including the early phase of infection, the onset of viremia and fever, and later stages—would provide a more comprehensive understanding of host immune responses and their relationship to LASV pathogenesis.

Several studies have reported that the L segment is a pathogenic factor in arenaviruses, such as the Pichinde virus, lymphocytic choriomeningitis virus, and ML-29 [29–32]. McLay et al demonstrated that 4 amino acid substitutions in the C-terminal domain of the Lp (T1808A, L1839V, N1889D, and N1906D) attenuated Pichinde virus in vivo by reducing replication efficiency [29]. In our study, LF2384 showed slightly lower replication than LF2350, suggesting that pathogenicity in vivo may not correlate directly with replication capacity. The minigenome reporter assay indicated higher RdRp activity for LF2350 than for LF2384, consistent with viral replication kinetics. Similar to findings in other viral systems, these results indicate that replication efficiency in vitro does not necessarily reflect pathogenicity in vivo [32–34]. In this context, unidentified functions of the LASV Lp may contribute to differences in pathogenicity. Previously, guinea pig-adapted LASV strains were shown to carry 2 amino acid substitutions—N1221D in Lp and I228V in NP—although their functional roles remain unclear [35]. Both LF2350 and LF2384 possess D at position 1221 in Lp, indicating that this site is not associated with their differential pathogenicity.

The crystal structure of LASV Lp was recently reported, and putative functional domains were identified [36]. We found 121 amino acid differences between LF2384 and LF2350 Lps, distributed across all predicted domains (Table 1 and Supplementary Figure 2). Despite these differences, amino acid identity across these domains exceeds 90%, indicating that multiple regions of the Lp ORF may contribute to pathogenicity. Determining the specific region responsible for pathogenicity would require the characterization of rLASVs with individual amino acid substitutions between the 2 strains. However, the high number of substitutions presents a challenge. Combined with the transcriptional profiling of immune-related genes in infected guinea pigs, further transcriptomic analyses using recombinant chimeric LASVs may help clarify the contribution of Lp to pathogenicity.

Notably, previous studies using the prototypic LASV strain Josiah in guinea pig models have suggested that the S segment, rather than the L segment, is the primary determinant of pathogenicity [37]. That study concluded that the pathogenicity of the Josiah strain in guinea pigs was mainly associated with the S segment. However, differences in viral strains, host models, and infection routes between our study and prior research may explain these divergent findings. Further investigations are necessary to elucidate the direct or indirect roles of the LF2384 Lp or the Josiah S segment in mediating interactions with host factors in guinea pigs.

Compared to mice, rats, and hamsters, guinea pigs lack well-established reagents, tools, and resources for immunological research [38]. This limitation hindered the depth of our analyses, particularly in exploring the relationship between Lp and host immune responses. Developing new reagents and tools for guinea pig models will be essential to enable more comprehensive investigations of LASV immunopathogenesis.

## Supplementary Material

jiaf323_Supplementary_Data

## Supplementary Data

Supplementary materials are available at *The Journal of Infectious Diseases* online (http://jid.oxfordjournals.org/). Supplementary materials consist of data provided by the author that are published to benefit the reader. The posted materials are not copyedited. The contents of all supplementary data are the sole responsibility of the authors. Questions or messages regarding errors should be addressed to the author.

## Notes

***Acknowledgment.*** The authors thank the staff of the Animal Resources Center at the University of Texas Medical Branch for their support.

***Data availability.*** All data are available in the main text or the Supplementary Materials. Sequences have been deposited in GenBank under accession numbers PP826286, PP826287, PP826288, and PP826289.

***Financial support.*** This work was supported by the National Institutes of Health (grant numbers K99AI156012 and R00AI156012 to J. M., and U01AI151801 to S. P.); Internal fund at the Institute for Human Infections and Immunity (to J. M.); and the John S Dunn Foundation (to S. P.).
