# Supplementary material for: Identification of Factors Contributing to Pathogenic Variability Among Lassa Virus Strains Using the Guinea Pig Model and Reverse Genetics
Source: J Infect Dis. 2025 Jun 21;232(5):e839–48. doi: 10.1093/infdis/jiaf323 (PMC12614964; doi:10.1093/infdis/jiaf323)
Supplement: jiaf323_Supplementary_Data [file jiaf323_supplementary_data.zip › FigS1-S3 JID.docx]

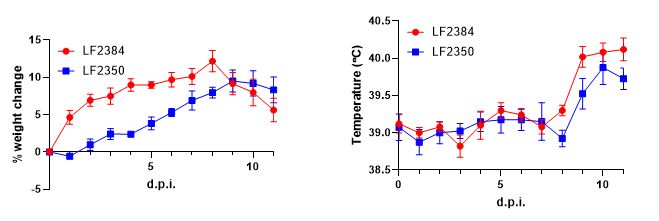


**Fig. S1 Body weight and temperature change in guinea pigs infected with 10^4^ PFU of LASV LF2384 or LF2350.**

Hartley guinea pigs were inoculated with 10^4^ PFU of LASV LF2384 or LF2350 intraperitoneally. Body weight (left) and temperature (right) were measured daily until 11 d.p.i. The means and standard errors were plotted. The number of animals were n=5 for LASV LF2384 and n=4 for LASV LF2350 since one guinea pig died after virus inoculation and blood sampling. Organ and blood samples was collected for CBC, blood clinical chemistry, virus dissemination, and transcription analyses.


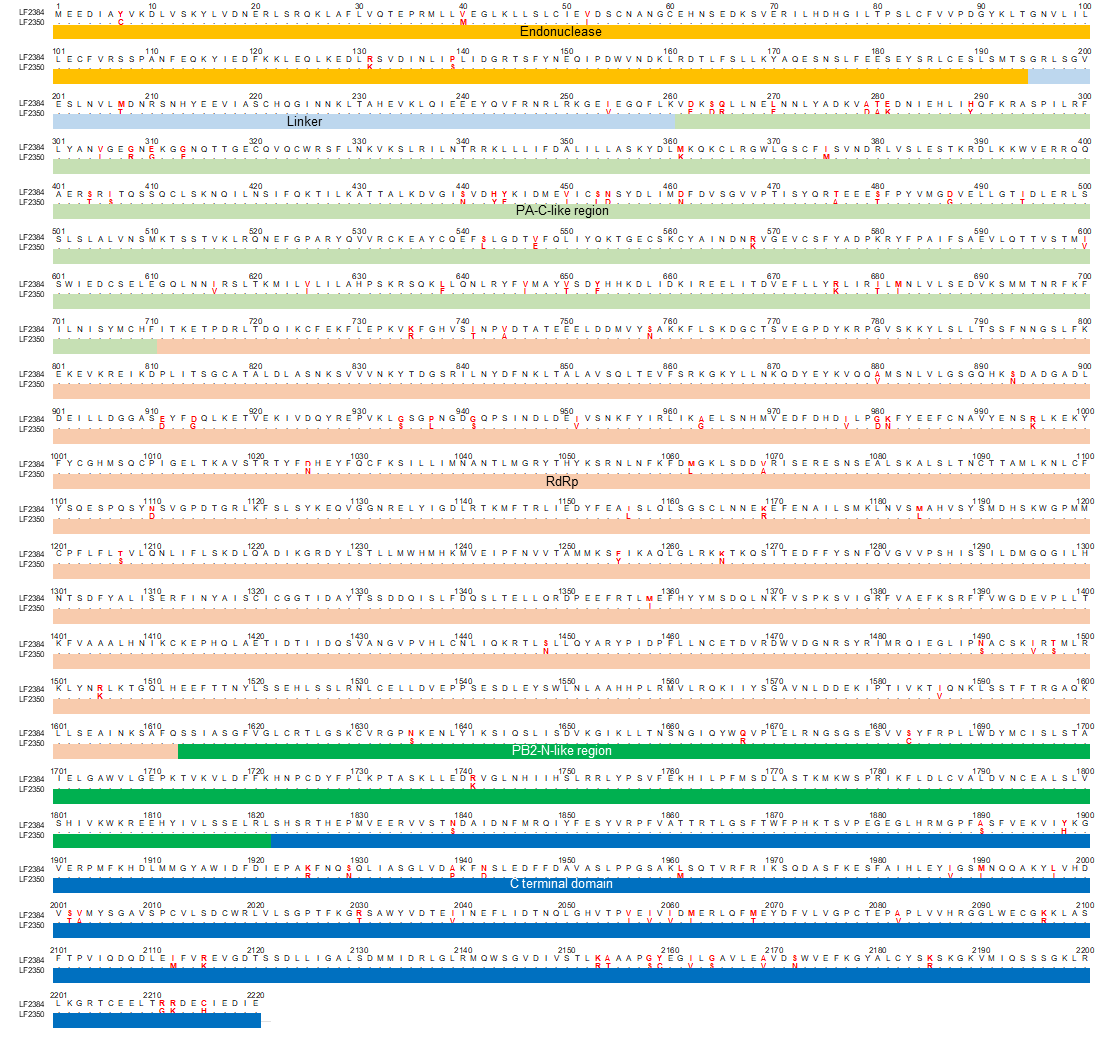


**Fig. S2 Comparison of amino acid sequences of L protein between LF2384 and LF2350.**

Amino acid sequences of the L protein of LASV LF2384 (PP826289) and LF2350 (PP826287) were aligned. Orange represents the endonuclease domain, light blue represents the linker domain, light green represents the PA-C-like domain, light pink represents the RdRp domain, green represents the PB2-N-like domain, and blue represents the C-terminal domain. Different amino acid residues between LF2384 and LF2350 are indicated by red letters.

**
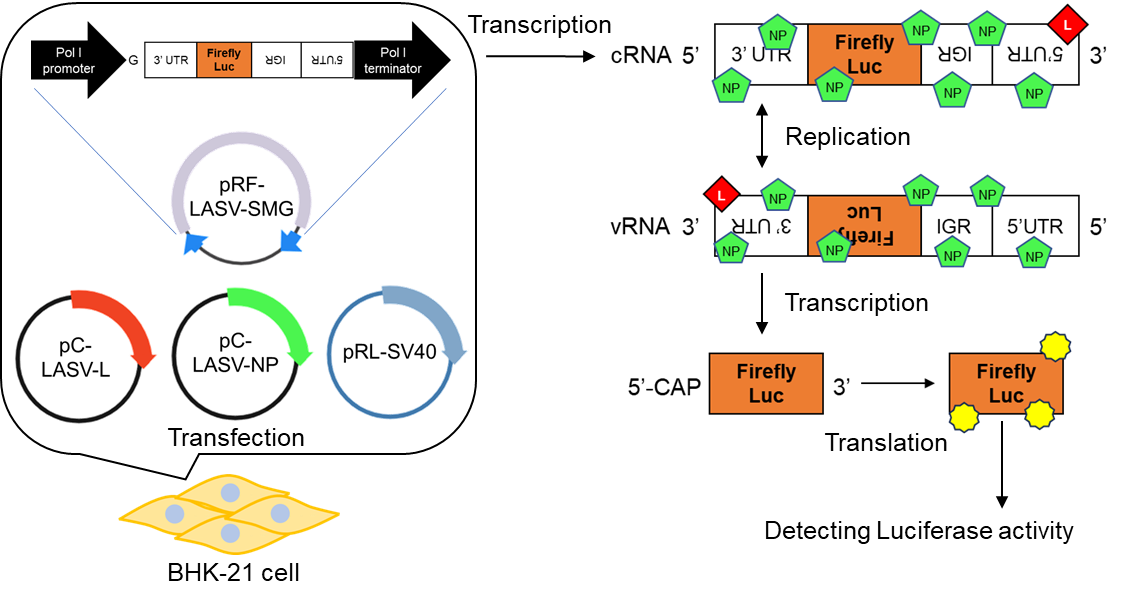
Fig. S3 Schematic diagram of the LASV minigenome assay.**

A diagram of the LASV S segment minigenome plasmid (pRF-LASV-SMG) and its replication, transcription, and translation mechanisms are shown. The cDNA fragment containing the S 3’-untranslated region (UTR), Firefly luciferase (Fluc) open reading frame, intergenic region (IGR) of S segment, and the S 5’-UTR in the anti-genomic sense is inserted between the murine RNA polymerase I (Pol-I) promoter and terminator. An additional deoxyguanosine is inserted between Pol-I promoter and the S 3’-UTR. pRF-LASV-SMG plasmid is transcribed by Pol-I to generate cRNA in BHK-21 cells. The cRNAs are encapsidated with the NP and the vRNAs are produced in the presence of L. The encapsidated vRNA is transcribed into a reporter-gene mRNA. Finally, the transcribed mRNA is translated by the host to produce Fluc. Renilla luciferase (Rluc) expressed by pRL-SV40 were measured as transfection control.
