# Supplementary material for: Identification of Factors Contributing to Pathogenic Variability Among Lassa Virus Strains Using the Guinea Pig Model and Reverse Genetics
Source: J Infect Dis. 2025 Jun 21;232(5):e839–48. doi: 10.1093/infdis/jiaf323 (PMC12614964; doi:10.1093/infdis/jiaf323)
Supplement: jiaf323_Supplementary_Data [file jiaf323_supplementary_data.zip › Supplementary information.docx]

Supplementary information

Supplementary methods- clinical scoring and endpoint criteria

The animals were observed daily, and their health statuses, temperatures, and weights were documented. If animals were found with clinical signs of disease, monitoring increased in frequency up to twice per day. The clinical signs were judged based on the manner shown below.

**Visual inspection**

Visual inspections for apparent illness, paralysis and death were performed daily for all animals and health statuses were recorded. Representative clinical signs were scruffy coat, hunched, and vomiting. If the guinea pigs have ruffled hair on their back, they were determined to be “Scruffy coat”. Guinea pigs with round backs were diagnosed as “hunched”.

**Body temperature**

A DAS-8027 transponder was implanted subcutaneously in the dorsal area of each animal between the scapulae using a trocar needle assembly provided with the chip. Body temperatures were collected every morning. The accepted normal range for guinea pig body temperature is 38.0°C– 39.5°C. If the body temperatures of guinea pigs were above 39.5°C, they were determined to be febrile. Guinea pigs whose body temperature were below 38.0°C after continuous fever were determined to be hypothermia.

**Endpoint criteria**

Animals with more than 20% of body weight loss, animals that were paralyzed or prostrated and unable to eat or drink, or those that were moribund (unable to move, unresponsive, unconscious), whichever occurs first, were euthanized with CO_2_ followed by thoracotomy.
